# Climate temperature and seasonal influences on the prevalence of temporomandibular disorders in South Korea

**DOI:** 10.1038/s41598-024-61829-2

**Published:** 2024-05-14

**Authors:** Yeon-Hee Lee, Jin-Woo Chung

**Affiliations:** 1 Department of Orofacial Pain and Oral Medicine, College of Dentistry, Kyung Hee University Dental Hospital, Kyung Hee University, #613 Hoegi-dong, Dongdaemun-gu, Seoul, 02447 South Korea; 2 Department of Oral Medicine and Oral Diagnosis, Seoul National University School of Dentistry, #101 Daehak-ro, Jongno-gu, Seoul, 03080 South Korea

**Keywords:** Seasonal, Winter, Temporomandibular disorder, Climate, Temperature, Prevalence, Diseases, Health care, Medical research, Risk factors

## Abstract

This study aimed to explore seasonal variations in temporomandibular disorder (TMD) prevalence in South Korea, utilizing nationwide population-based big data. Data corresponding to the Korean Standard Classification of Diseases code of K07.6, which identifies TMD, were extracted from the Health Insurance Review and Assessment Service online platform for the period from 2010 to 2022. Additionally, we integrated these data with climate temperature records from the Korean Meteorological Administration. We subsequently conducted a statistical analysis of TMD patient data on a monthly and seasonal basis over the past 13 years to assess prevalence. Over the past 13 years, the number of TMD patients in Korea has steadily increased. The prevalence of TMD rose from 0.48% (224,708 out of a total population of 50,515,666) in 2010 to 0.94% (482,241 out of a total population of 51,439,038) in 2022, marking a 1.96-fold increase. Among children under 10 years of age, no significant differences were observed in TMD prevalence between boys and girls. However, a distinct female predominance emerged after the age of 10, with an average female-to-male ratio of 1.51:1. The peak prevalence of TMD occurred in individuals in their 20 s, followed by adolescents in their late 10 s. The majority of TMD patients were concentrated in Seoul and Gyeonggi province, with metropolitan areas accounting for 50% of the total patient count. Seasonally, TMD patient numbers showed no significant increase in winter compared with spring or summer. The temperature difference, defined as the absolute difference between the highest and lowest temperatures for each month, showed a positive correlation with TMD patient counts. A greater temperature difference was associated with higher patient counts. The strongest correlation between temperature differences and TMD patient numbers was observed in winter (r = 0.480, p < 0.01), followed by summer (r = 0.443, p < 0.01), and spring (r = 0.366, p < 0.05). Temperature differences demonstrated a significantly stronger correlation with the increase in the number of TMD patients than absolute climate temperatures. This aspect should be a key consideration when examining seasonal trends in TMD prevalence in South Korea.

## Introduction

South Korea is renowned for its pronounced seasonal diversity encompassing spring, summer, fall, and winter. Spring, observed from March to June, sees temperatures ranging between 8 and 20 °C, coinciding with the blossoming of flora and the emergence of leaves. Although it typically is warm and agreeable, intermittent cold snaps may be accompanied by brisk winds. Summer, spanning from July to August, experiences temperatures surging between 25 and 35 °C accompanied by escalated humidity, often resulting in sultry and occasionally rainy conditions during the monsoon season^1^. This season may encounter strong winds and heavy rainfall due to the influence of typhoons. Fall, extending from September to mid-November, boasts mild temperatures ranging from 10 to 20 °C, characterized by clear and pleasant weather. Finally, winter spanning from December to February with witnessed temperatures plunging below freezing temperatures, ranging from − 6 to 3 °C, particularly in inland and mountainous areas, with numerous regions experiencing subzero temperatures. Frequent snowfall is common and is compounded by varying humidity levels that contribute to a further reduction in perceived temperatures^2^. Notably, South Korea’s climate presents a unique contrast, with a difference in temperature of over 35 °C between winter and summer.

Temporomandibular disorders (TMD) include various conditions affecting the temporomandibular joint (TMJ) and the associated masticatory muscles, bones, and tissues^3^. The etiology of TMD is multifaceted, potentially involving muscle or cartilage damage around the TMJ, TMJ instability, muscle tension due to psychological stress, malocclusion, macrotrauma, microtrauma, and systemic disorders, such as rheumatism or growth issues during childhood or adolescence^4,5^. While prevalence rates may vary based on sex, age, and geographical location, reports indicate that most individuals experience TMD-related symptoms at least once in their lifetimes^6^. TMD is highly prevalent worldwide, with an overall prevalence of approximately 31% in adults and 11% in children/adolescents^7^. Notably, it tends to be a 2–4 times higher prevalence among women compared to men, particularly in their 20 s and 40 s^8,9^.

The signs and symptoms of TMD are diverse and contribute to its diagnosis. Key symptoms include pain or discomfort in the TMJ area; TMJ noises such as clicking, popping, and crepitus; sensation of jaw locking or restricted mandibular movement; deformities or swelling around the TMJ; muscle stiffness; ear pain; tinnitus; neck pain; and headaches^10^. These symptoms significantly affect daily life and restrict activities such as eating, speaking, and facial expressions. Accurate diagnosis and appropriate treatment planning are crucial for effective management of TMD. Additionally, persistent TMD symptoms may be accompanied by sleep disturbances or psychological issues^11,12^. Hence, understanding the nature of TMD and addressing its diagnosis, treatment, management, and prevention is imperative.

The prevailing belief that musculoskeletal disorders are influenced by weather factors is well-established. TMD, a key musculoskeletal condition of the oromaxillofacial region, exemplifies this, as patients often report exacerbated pain during cold and rainy conditions^6^. Pain, a critical symptom of musculoskeletal disorders, can fluctuate in severity in response to environmental variables such as temperature, pressure, and humidity^13^. Among individuals suffering from these disorders, the correlation between perceived climatic conditions and pain suggests that personal experiences of the climate may effectively indicate the need for cold exposure mitigation and protective measures. For TMD patients, prevention strategies, including the use of appropriate clothing and moist heat therapy, are often both cost-effective and simple to implement^14,15^. However, the role of cold exposure as a direct causative factor for musculoskeletal conditions remains uncertain^16^. Moreover, the impact of seasonality and temperature on the incidence of TMD has yet to be explored in South Korea. Given the variability of seasons and climate temperatures related to the country’s topography, conducting region-specific analyses is essential. Therefore, this study aims to investigate the effects of the winter season and low temperatures to assess the objectivity and scientific validity of the prevailing beliefs held by both patient and clinician communities.

Historically, TMD has been classified with various systems, such as the Research Diagnostic Criteria for Temporomandibular Disorders (RDC/TMD) and the Diagnostic Criteria for Temporomandibular Disorders (DC/TMD), which play significant roles in defining and categorizing TMD. The RDC/TMD, developed in the 1990s, was among the initial comprehensive systems used for diagnosing TMD^17^. It is categorized based on clinical and imaging criteria and divided into groups such as myofascial pain, internal derangement, and arthralgia. Subsequently, the DC/TMD introduced by the International RDC/TMD Consortium Network in 2014 refined and updated the classification system^10^. It expanded the diagnostic categories, incorporated more specific assessment methods, and integrated both physical and psychosocial factors, emphasizing a more comprehensive approach to diagnosing and managing TMD. In South Korea, TMD specialists and general dentists diagnose patients based on the international TMD diagnostic criteria. National data in South Korea are categorized under the overarching term temporomandibular disorder (K07.6) and its subcategories (K07.60-K07.69). In this study, we exclusively focused on the 4-digit code ‘K07.6’ from the Korean Standard Classification of Diseases (KCD). Codes associated with congenital disorders, growth abnormalities, cysts, and both benign and malignant tumors, which could lead to TMD-like signs and symptoms, were deliberately excluded. This exclusion was specifically aimed at concentrating the analysis on typical and representative cases of TMD.

Research on TMD using South Korea’s healthcare big data and national population-based data is limited. To date, associations between various factors such as region, race, and sex and TMD occurrence have been reported^8,18,19^. During winter, the human body adapts to cold weather and attempts to regulate the internal temperature, increase muscle contraction, and decrease oxygen supply to the muscles, resulting in increased muscle pain or stiffness^20–22^. Arthritic pain can also increase during cold winters and at low temperatures as opposed to during summer and warm temperatures. A recent systematic review of osteoarthritis pain reported a negative correlation between temperature and pain severity^23^. However, the specific relationship between TMD pain and weather conditions remains ambiguous. Our primary hypothesis is that the season of winter and low climate temperatures are associated with an increase in TMD patient counts. Moreover, this study aims to explore the relationship between the difference in minimum and maximum temperatures and TMD prevalence. Utilizing nationwide population-based data from the Health Insurance Review and Assessment Service (HIRA), also known as National Health Insurance data, this study investigates seasonal fluctuations in TMD prevalence in South Korea. According to data from the Ministry of Public Administration and Security as of March 2024, the total population of South Korea was 51,293,934, with Seoul (the capital) accounting for 18.29%, Gyeonggi Province for 26.60%, and the combined Seoul–Gyeonggi metropolitan area for 44.89%. This study also aims to investigate whether the distribution of TMD patients correlates with the general population distribution.

## Methods

The research protocol for this study adhered to the principles of the Declaration of Helsinki and received approval from the Institutional Review Board of Kyung Hee University Dental Hospital, Seoul, South Korea (KHD IRB, IRB No-KH-DT23043).

### Study population

HIRA serves as a nationally representative healthcare big data repository that collects billing data throughout the reimbursement process for healthcare service providers. The universal coverage system contains extensive and comprehensive information regarding medical services encompassing treatments, medications, procedures, and diagnoses for around 50 million beneficiaries covered under South Korea’s universal healthcare system. The HIRA dataset, which constitutes a substantial repository within the healthcare sector, has a significant potential for creating value across various domains. This aids in enhancing the efficiency of healthcare delivery systems while maintaining high-quality care, offering support for specific interventions, and furnishing crucial information to prevent or monitor side effects. Leveraging HIRA data for research is imperative to unlock its potential in understanding symptoms and managing, treating, and addressing activities related to patients with TMD.

The 4-digit code ‘K07.6’ of KCD in HIRA serves as an umbrella term for ‘temporomandibular joint disorders’. The detailed subcategories encompassed by K07.6 include internal derangement of TMJ, snapping jaw, recurrent dislocation and subluxation of TMJ, degenerative joint disease of TMJ, degenerative arthritis of TMJ, Costen’s sydrome, pain in TMJ, stiffness of TMJ, TMJ pain and dysfunction syndrome, masticatory muscle disorders. Only data related to the K07.6 code were analyzed. Since the HIRA only releases data from 2010 onwards, and the data from 2022 is the most current, we attempted to analyze as much data as possible from the period between 2010 and 2022. To examine long-term trends, all cases classified as K07.6 in the HIRA dataset over a 13-year period from 2010 to 2022 were analyzed. To assess the short-term data distribution over a 1-year period, the most recent data from 2022 were utilized.

Atypical cases that can cause pain or discomfort in the TMJ region, such as congenital diseases of the TMJ or benign or malignant tumors of the TMJ, were excluded from this study. For example, in the KCD, the following codes were excluded: C41.1 (malignant neoplasm of mandible), C71.2 (malignant neoplasm of temporal lobe), D10.3 (benign neoplasm of oral cavity), D16.5 (benign neoplasm of mandible), K10.8 (condylar hyperplasia or hypoplasia), Q67.0 (facial asymmetry), and S02.6 (fracture of the angle of the mandible).

### Data acquisition

All the data used in this study were obtained from officially accredited sources in the public domain. Information regarding Korean patients’ health statuses, medical providers, medical expenses, utilization rates, and medical service summary statistics were accessed through the Healthcare Big Data Open System (http://opendata.hira.or.kr). In addition, monthly temperature data over the past 13 years were obtained from the National Climate Data Center of the Korea Meteorological Administration (https://data.kma.go.kr). For the mean temperature, the lowest temperature, and the highest temperature for each month, data sources provided by the country on the online platform were used. The Korea Meteorological Administration typically determines mean, lowest, and highest temperatures using a combination of ground-based weather stations, remote sensing technology, and other data collection methods^24^. The ‘temperature difference’ in this study refers to the absolute value of the difference between the highest and lowest temperatures for each month, calculated by subtracting the monthly lowest temperature from the monthly highest temperature. To determine the ratio of TMD patients to the total population, national total population data was also used. The total population of South Korea for the past 13 years, spanning from 2010 to 2022, was sourced from the online National Population Statistics portal (https://jumin.mois.go.kr). These data sets were chronologically arranged by year and month, merged, and subjected to statistical analysis.

### Statistical analysis

Data were analyzed using the Statistical Package for Social Sciences (SPSS) for Windows (version 26.0; IBM Corp. Armonk, NY, USA). Descriptive statistics were reported as mean ± standard deviation or numbers with percentages, as appropriate. The t-test was used to compare the means between the two groups. Analysis of variance and Tukey’s post-hoc tests were used to compare the parameter values for the four seasons (spring, summer, fall, and winter) and for the 12 months from January to December. Spearman’s correlation analysis was conducted to explore the correlation between two parameters. The closer the absolute value of the correlation coefficient (r) is to 1, the stronger the correlation, whereas values closer to 0 indicate a weaker correlation. Statistical significance was set at a two-tailed p-value < 0.05.

### Ethics declaration

The protocol for this study was exempt from review by the Institutional Review Board of Kyung Hee University Dental Hospital (KHD IRB, IRB No-KH-DT23043).

## Results

### Prevalence and the number of patients with TMD (2010–2022)

**Figure 1:**
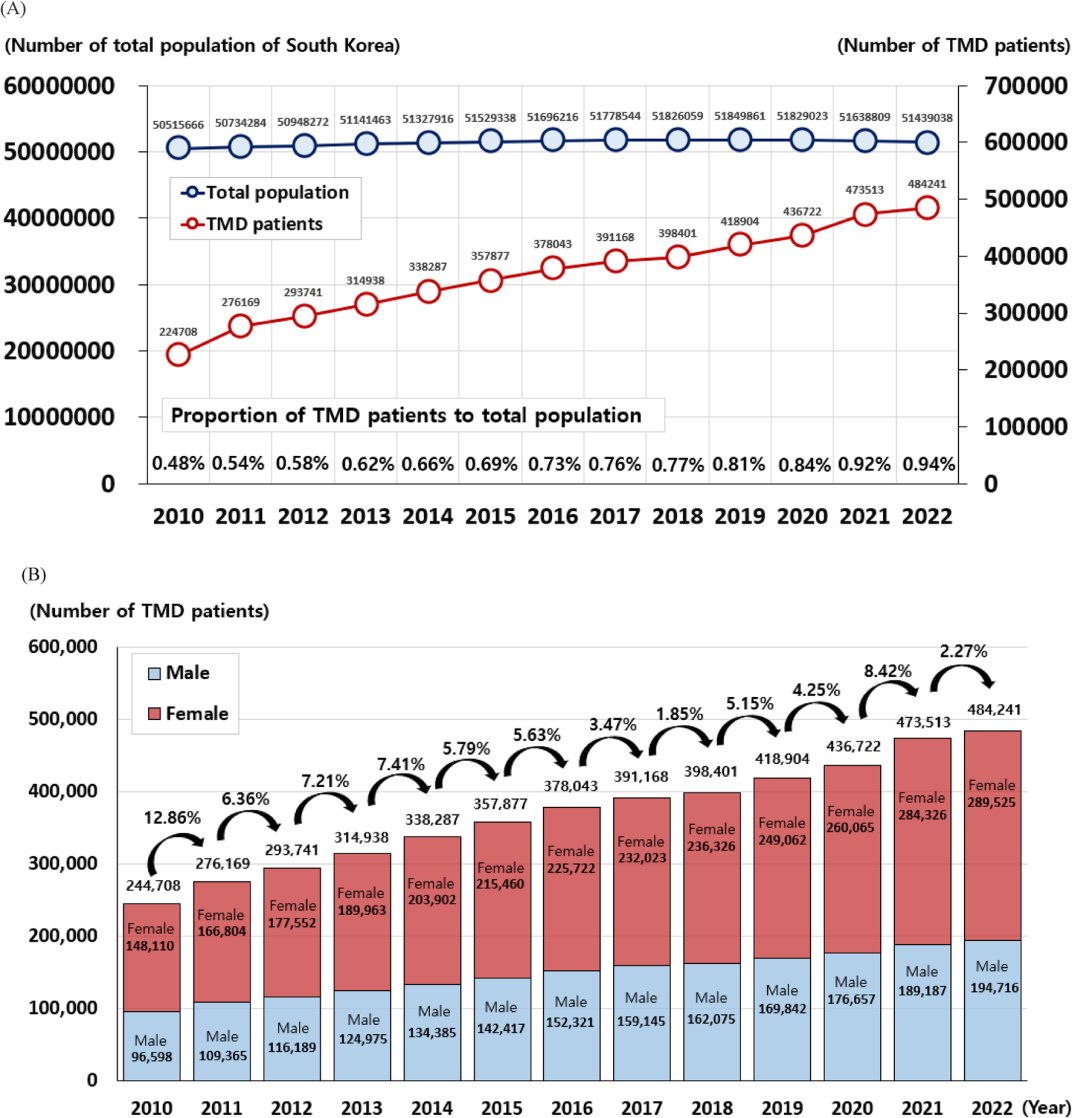
Increase in the number of TMD patients over the past 13 years (2010–2022). (**A**) The ratio of TMD patients to the total population over the past 13 years. (**B**) Changes in the number and increase rate of TMD patients over the past 13 years.

By analyzing health data from January 2010 to March 2022 published by the HIRA we investigated the number of patients (prevalence) who visited hospitals due to TMD in Korea. In 2022, the total population of Korea was 51.74 million, and the number of patients who visited hospitals and clinics due to TMD was 484,241; accounting for 0.94% of the total Korean population (Fig. 1A). Despite minimal fluctuations in the population of South Korea, from 50,515,666 in 2010 to 51,439,038 in 2022, with a peak at 51,849,861 in 2018, the overall population growth over these 13 years was approximately 1.02-fold. In stark contrast, the prevalence of TMD patients demonstrated a consistent upward trend throughout the same period. The absolute number of TMD patients escalated from 224,708 in 2010 to 482,241 in 2022, marking an increase of 97.89%. Correspondingly, the proportion of TMD patients relative to the total population sharply increased from 0.48% in 2010 to 0.94% in 2022, marking a 1.96-fold increase.

The period with the largest increase in the number of TMD patients over the 13 years studied was 2010–2011, with an increase of 12.86%. The second period with the largest increase was 2020–2021, with an increase of 8.42% (Fig. 1B). Further investigation is needed to determine whether the increase in the number of patients with TMD between 2020 and 2021 is correlated with the COVID-19 pandemic.

The number of females with TMD increased from 148,110 in 2010 to 289,525 in 2022, marking a surge of 95.48%. Among males, the increase was 101.57%, from 96,598 in 2010 to 194,716 in 2022. Consequently, the growth rate of TMD patients in males was 6.09% higher than that in females (Fig. 1B).

### Prevalence of TMD by age group (2010–2022)

An analysis of data from 2010 to 2022 revealed distinct patterns in the prevalence of TMD. The peak prevalence was noted among individuals in their 20 s, with the female-to-male ratio averaging 1.51:1 across all age groups. Notably, in children under 10 years, the prevalence of TMD did not statistically significantly differ between sexes, indicating an absence of the female predominance typically observed in older TMD patient populations. Conversely, in age groups older than 10 years, females consistently exhibited a significantly higher prevalence of TMD compared to males (Table 1).

**Table 1:** Distribution of TMD patients by age group and sex over the past 13 years (2010–2022).

Age group (5 years interval)	Total	p-value^a^	Post-hoc^a^	Male	Female	p-value^b^
Age group 1 (< 5 years)	316.15 ± 73.11	< 0.001***	Age group 1, 2 < 16, 17 < 3, 13, 14, 15 < 12 < 8, 9, 10, 11 < 7 < 6 < 4 < 5	153.85 ± 39.61	162.31 ± 35.22	0.574
Age group 2 (5-9 years)	1741.85 ± 127.99			919.54 ± 71.86	822.31 ± 67.89	0.254
Age group 3 (10-14 years)	15,324.54 ± 1409.23			6185.69 ± 502.33	9138.85 ± 949.30	< 0.001***
Age group 4 (15-19 years)	48,946.62 ± 3357.89			22,474.46 ± 1414.74	26,472.15 ± 2095.89	< 0.001***
Age group 5 (20-24 years)	55,780.15 ± 10,542.56			23,671.17 ± 5025.04	32,109.15 ± 5568.29	< 0.001***
Age group 6 (25-29 years)	46,315.23 ± 11,147.79			18,260.46 ± 4806.20	28,054.76 ± 6369.04	< 0.001***
Age group 7 (30-34 years)	32,939.31 ± 6111.81			12,731.23 ± 2804.68	20,208.08 ± 3325.49	< 0.001***
Age group 8 (35-39 years)	27,568.00 ± 5059.66			10,197.46 ± 2128.29	17,370.54 ± 2943.74	< 0.001***
Age group 9 (40-44 years)	25,459.54 ± 5063.13			9314.46 ± 1763.35	16,145.08 ± 3309.48	< 0.001***
Age group 10 (45-49 years)	24,282.23 ± 6107.95			8961.02 ± 2139.51	15,321.23 ± 3973.65	< 0.001***
Age group 11 (50-54 years)	23,432.54 ± 5744.97			8666.38 ± 2125.13	14,766.15 ± 3625.42	< 0.001***
Age group 12 (55-59 years)	20,935.54 ± 6659.44			8014.46 ± 2539.32	12,921.08 ± 4124.13	< 0.001***
Age group 13 (60-64 years)	16,972.85 ± 7150.90			6713.31 ± 2751.53	10,259.54 ± 4401.49	0.0007***
Age group 14 (65-69 years)	13,419.85 ± 5062.56			5379.08 ± 2049.93	8040.76 ± 3017.80	< 0.001***
Age group 15 (70-74 years)	10,412.00 ± 3067.31			4106.77 ± 1366.7	6305.23 ± 1705.56	< 0.001***
Age group 16 (75-79 years)	7231.06 ± 2546.75			2647.31 ± 1058.51	4583.69 ± 1490.62	< 0.001***
Age group 17 (≥ 80 years)	4823.14 ± 2252.60			1615.15 ± 854.43	3207.85 ± 1399.03	< 0.001***

^a^The results were obtained through ANOVA and Tukey’s post-hoc analyses, with comparisons of mean values performed across age groups 1 to 17.

^b^The results were analyzed using t-tests; comparisons of mean values by sex were conducted. Statistical significance was set at p < 0.05, ***p < 0.001.

The significant results are shown in bold.

The prevalence of TMD varied significantly across age groups, and the increase in the number of TMD patients did not correlate simply or unidirectionally with age increments or decrements. The highest prevalence of TMD was observed in age group 5 (20–24 years), with an average of 55,780.15 ± 10,542.56 individuals. The next highest prevalence was in age group 4 (15–19 years), where the number of cases averaged 48,946.62 ± 3357.89. Examining the prevalence of TMD in descending order, the age groups with the lowest prevalence were age group 1 (under 5 years) with 316.15 ± 73.11 individuals and age group 2 (5–9 years) with 1741.85 ± 127.99 individuals. The next lowest prevalence was observed in age group 17 (80 years and older) with 4823.08 ± 2252.60 individuals, followed by age group 16 (75–79 years) with 7231.06 ± 2546.75 individuals (p < 0.001). While age group 4 (15–19 years) displayed the second highest prevalence, age group 3 (10–14 years) showed a relatively lower prevalence rate with 15,324.54 ± 1409.23 individuals, ranking seventh among the 17 age groups examined (Table 1).

### Peak prevalence and female-to-male ratio in 2022

**Figure 2:**
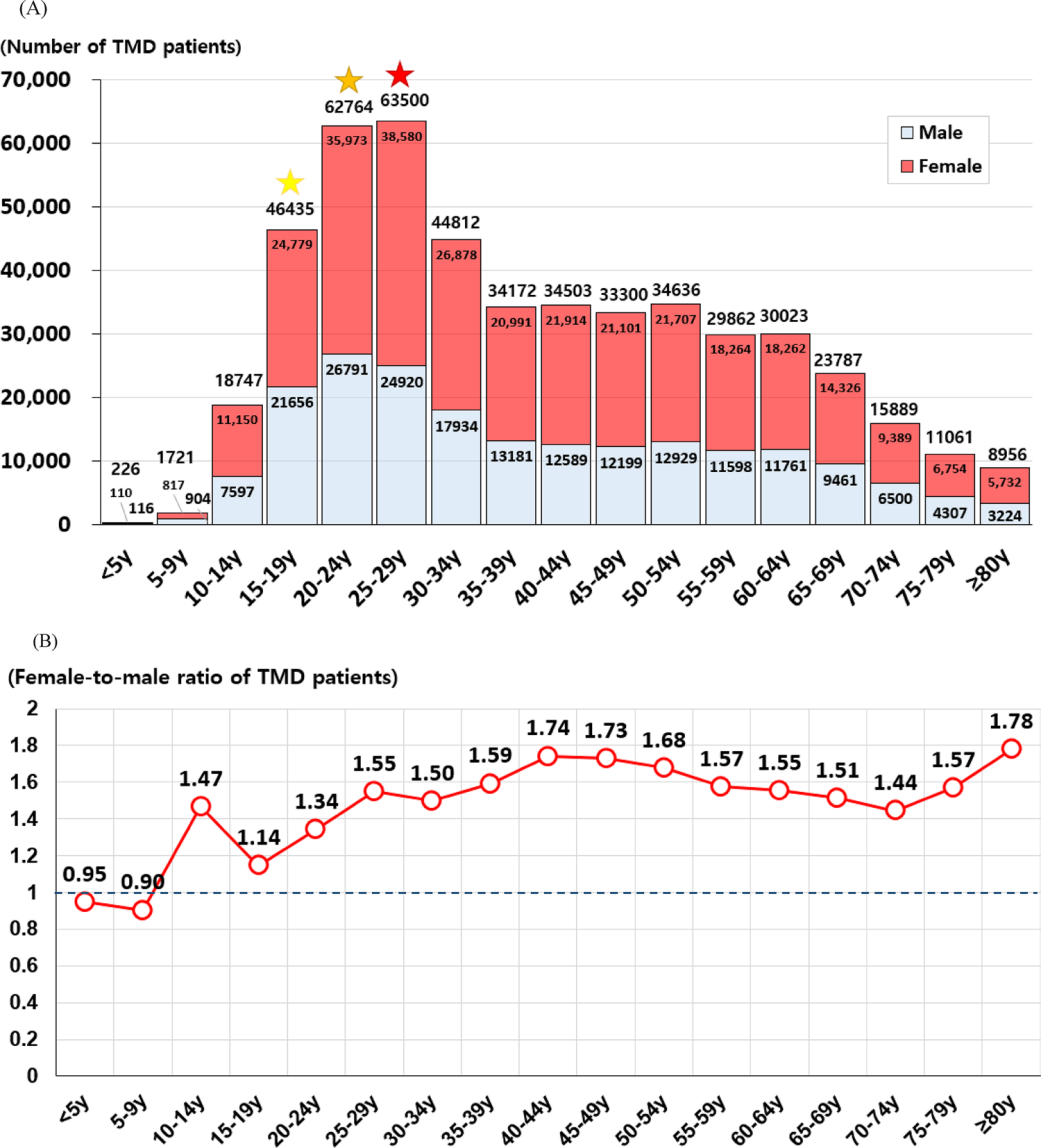
Distribution of TMD patients by age and sex in 2022. (**A**) Distribution of TMD patients by age group with a 5-year age gap in 2022. Red star: 1st in number of TMD patient; orange star: 2nd in number of TMD patient; yellow star: 3rd in number of TMD patient. (**B**) Female-to-male ratio of TMD patients with a 5-year age interval in 2022. *TMD* temporomandibular disorder.

To analyze the distribution of TMD patients by age and sex within a one-year period, we utilized data from 2022, the most recent year in our dataset spanning the past 13 years. Patients who visited the hospital in 2022 were categorized into seventeen age groups, segmented at 5-year interval. The age group with the highest number was age group 6 of 25–29 years old (63,500 individuals, 13.11%), followed by age group 5 of 20–24 years old (62,764 individuals, 12.96%), age group 4 of 15–19 years old (46,435 individuals, 9.59%), age group 7 of 30–34 years old (44,812 individuals), age group 11 of 50–54 years old (34,636 individuals), age group 9 of 40–44 years old (34,503 individuals), age group 8 of 35–39 years old (34,172 individuals), age group 10 of 45–49 years old (33,300 individuals), age group 13 of 60–64 years old (30,023 individuals), age group 12 of 55–59 years old (29,862 individuals), age group 14 of 65–69 years old (23,787 individuals), age group 3 of 10–14 years old (18,747, 3.87%), age group 15 of 70–74 years old (15,889 individuals), age group 16 of 75–59 years old (11,061 individuals), age group 17 of 80 + years old (8,956 individuals), age group 2 of 5–9 years old (1721 individuals), and age group 1 of < 5 years old (226 individuals); the frequency trends according to age group among all TMD patients were similar in both males and females (Fig. 2A).

Interestingly mid-to-late teens (15–19 years old) had the 3rd highest number of TMD patients out of a total of 17 age groups, while early teens (10–14 years old) ranked 12th. Additionally, TMD occurred even in children under the age of 10 years (1947 individuals, 0.40%), and in very elderly individuals aged over 80 years (8956 individuals, 1.85%) (Fig. 2A). Follow-up investigations are necessary to ascertain whether the prevalence of TMD will increase among individuals aged 80 or older in South Korea, which is projected to become a super-aged society next year.

### The female-to-male ratio across age groups in 2022

The overall female-to-male ratio among TMD patients visiting hospitals for treatment or management was 1.49:1 (in 2022). The female-to-male ratio of TMD patients who visited the hospital in 2022 was also calculated by dividing them into 17 age groups with (5-year age interval (Fig. 2B). Among the 17 age groups, males outnumbered females in the < 5 (0.95:1) and 5–9 (0.90:1) age groups. Excluding these two age groups, in all 15 age groups, TMD was more prevalent in women than in men (ranges 1.14–1.78:1). The highest ratio was 1.78 in the ≥ 80 age group. The lowest ratio was in the 15–19 age group, with a value of 1.14:1 (Fig. 2B).

### Distribution of TMD patients by type of healthcare institution in 2022

**Figure 3:**
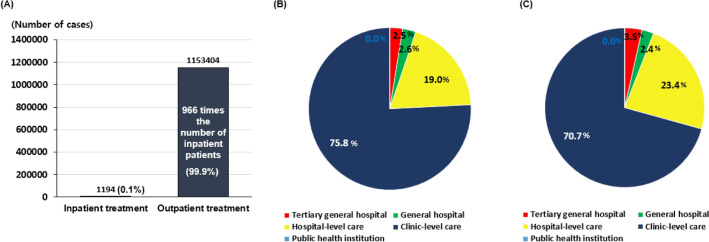
Distribution of TMD patients by type of healthcare institution in 2022. (**A**) Distribution of TMD patients between inpatient and outpatient settings. (**B**) Distribution of total medical expenses by type of healthcare institution (%), and (**C**) proportion of treatment days by type of healthcare institution (%).

In South Korea, the treatment of patients with TMD was conducted on an outpatient basis, and outpatients (99.9%) overwhelmingly outnumbered inpatients (0.1%) (Fig. 3A). Considering each type of healthcare institution, in the distribution of total medical expenses by type of healthcare institution, clinic-level care accounted for an overwhelmingly higher proportion (75.8%) than other types (Fig. 3B). Medical expenses for hospital-level care accounted for 19%, while general hospitals accounted for 2.6%, and tertiary general hospitals accounted for 2.5% of the total. The medical expenses of patients with TMD at public health institutions were 0.0%. Statistics on treatment days also showed a similar pattern to that of medical expenses, with clinic-level care accounting for an overwhelmingly large proportion (70.7%; Fig. 3C). This was followed by hospital care (23.4%), tertiary general hospitals (3.5%), general hospitals (2.4%), and public health institutions (0.0%).

### The distribution of the number of TMD patients by administrative region (2010–2022)

South Korea’s territory is divided into a total of 17 administrative regions. Specifically, South Korea comprises 9 provinces and 8 cities, including one special city and seven metropolitan cities. The nine provinces include Gyeonggi, Chungbuk, Chungnam, Gyeongbuk, Gyeongnam, Jeonbuk, Jeonnam, and Gangwon. The eight cities are Seoul (the special city), Incheon, Daejeon, Busan, Daegu, Gwangju, Ulsan, Jeju, and Sejong. We examined the distribution of TMD patients across the 17 administrative divisions of South Korea based on data provided by the HIRA.

**Figure 4:**
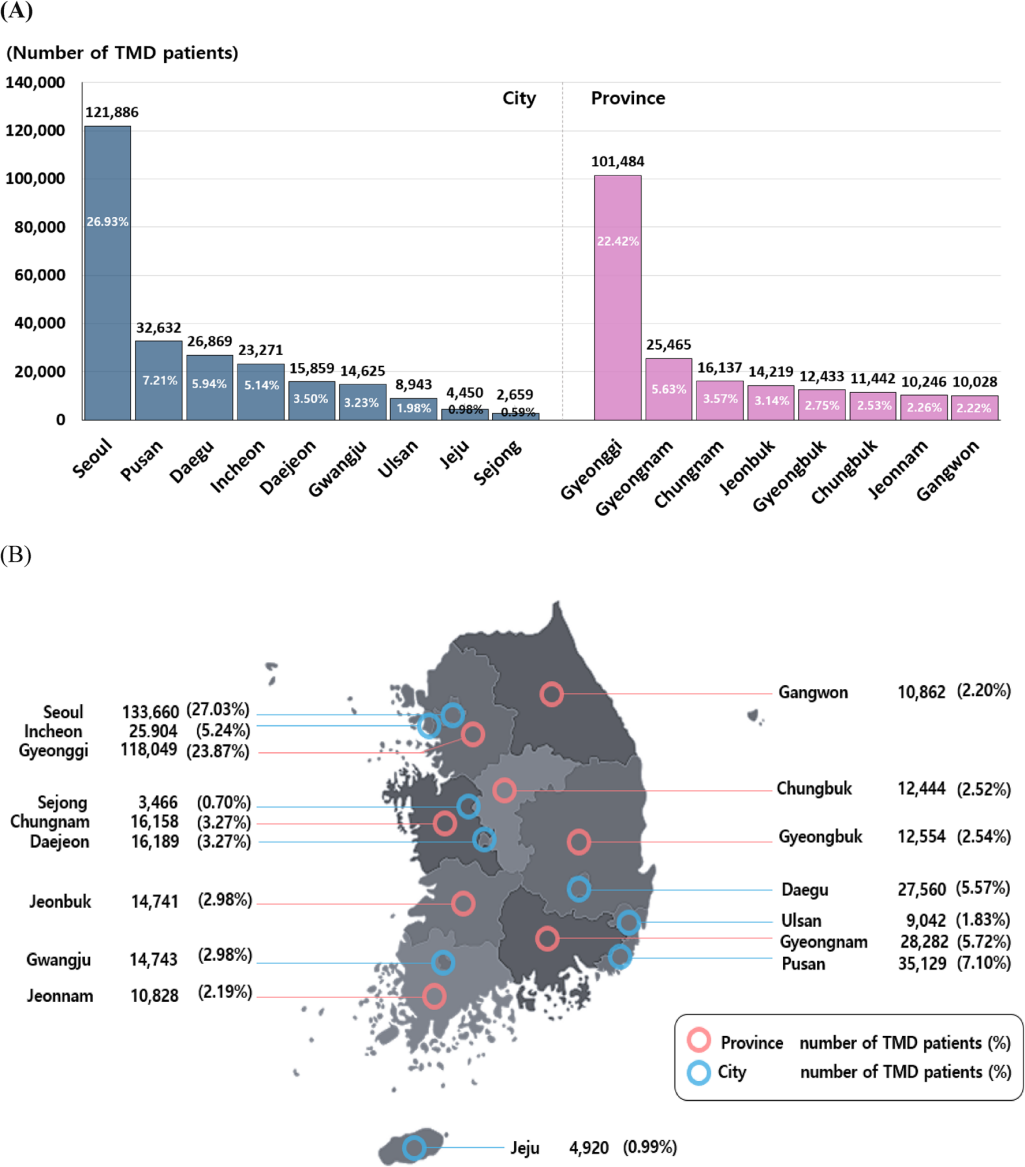
Distribution of TMD patients by administrative region in South Korea: analysis of 9 provinces and 8 cities. (**A**) Average distribution of TMD patients by administrative region in South Korea, 2010–2022. (**B**) Distribution of TMD patient numbers by administrative region in South Korea, 2022.

An analysis of the average number of TMD patients by administrative region from 2010 to 2022 was performed, revealing significant geographical variations in patient distribution across South Korea. At the city level, the distribution of TMD patients in descending order was as follows: Seoul (26.9%), Pusan (7.21%), Daegu (5.94%), Incheon (5.14%), Daejeon (3.50%), Gwangju (3.23%), Ulsan (1.98%), Jeju (0.98%), and Sejong (0.59%). At the province level, the distribution in descending order was Gyeonggi (22.42%), Gyeongnam (5.63%), South Chungnam (3.57%), Jeonbuk (3.14%), Gyeongbuk (2.75%), Chungbuk (2.53%), Jeonnam (2.26%), and Gangwon (2.22%). Seoul (the special city) and Gyeonggi province together accounted for 49.35% of all TMD cases from 2010 to 2022, with Seoul contributing 26.93% and Gyeonggi Province contributing 22.42% of the total cases (Fig. 4A). That is, the prevalence in the capital region, including Seoul and Gyeonggi, accounted for approximately 49.35% of the total, nearly half of the total occurrence.

During the one-year period of 2022, the distribution of TMD patients by administrative region was investigated. The distribution trend was similar to the results obtained from the 13-year data analysis. In 2022, the prevalence of TMD in the capital region, comprising Seoul City (27.03%) and Gyeonggi Province (23.87%), accounted for 50.9% of the total cases (Fig. 4B).

### TMD patient numbers and temperatures across the 12 months of the year (2010–2022)

Data on the number of TMD patients were obtained from the HIRA, and data on mean temperature, lowest temperature, and highest temperature were acquired from the Korea Meteorological Administration. Upon integrating and analyzing these datasets, the distribution of TMD patients from January to December did not exhibit statistically significant changes across the months (p = 0.974) (Table 2).

**Table 2:** Distribution of TMD patients and climate temperatures by month over the past 13 years (2010–2022).

Season	Month	Number of TMD patients	p-value^a^	Mean temperature (°C)	p-value^b^	Post-hoc^b^	Lowest temperature (°C)	p-value^c^	Post-hoc^c^	Highest temperature (°C)	p-value^d^	Post-hoc^d^	Temperature difference (°C)	p-value^e^	Post-hoc^e^
Spring	March	46,631 ± 10,996	0.974	**6.554 ± 1.619**	< 0.001***	1, 12 < 2 < 3, 11 < 4 < 5, 10 < 6, 9 < 7, 8	**2.138 ± 1.374**	< 0.001***	1 < 2, 12 < 3, 11 < 4 < 10 < 5 < 6, 9 < 7, 8	**11.838 ± 2.058**	< 0.001***	1, 12 < 2 < 3, 11 < 4 < 10 < 5, 9 < 6, 7, 8	**9.700 ± 1.111**	< 0.001***	7, 8 < 1, 12 < 2, 9, 11 < 3, 6, 10 < 4, 5
	April	45,930 ± 11,392		**12.350 ± 1.677**			**7.75 ± 1.418**			**17.783 ± 2.074**			**10.033 ± 0.817**		
	May	46,577 ± 11,644		**18.550 ± 0.914**			**13.767 ± 0.668**			**24.092 ± 1.400**			**10.325 ± 1.0567**		
	June	43,590 ± 10,368		**23.317 ± 0.678**			**19.233 ± 0.709**			**28.433 ± 0.899**			**9.200 ± 0.743**		
Summer	July	50,266 ± 12,519		**26.017 ± 1.159**			**23.05 ± 1.007**			**29.817 ± 1.408**			**6.767 ± 0.885**		
	August	49,292 ± 10,801		**26.742 ± 1.047**			**23.65 ± 0.950**			**30.625 ± 1.352**			**6.975 ± 0.966**		
Fall	September	45,528 ± 11,205		**22.017 ± 0.591**			**18.108 ± 0.643**			**26.625 ± 0.946**			**8.517 ± 0.958**		
	October	46,793 ± 11,659		**15.233 ± 1.008**			**10.683 ± 0.996**			**20.592 ± 1.187**			**9.908 ± 0.632**		
	November	48,370 ± 11,421		**7.567 ± 1.538**			**3.601 ± 1.736**			**12.217 ± 1.511**			**8.617 ± 0.878**		
Winter	December	48,718 ± 11,668		** − 0.6177 ± 1.747**			** − 4.358 ± 1.643**			**3.575 ± 1.907**			**7.933 ± 0.641**		
	January	47,164 ± 10,510		** − 2.325 ± 2.156**			** − 6.067 ± 2.037**			**2.017 ± 2.385**			**8.083 ± 0.769**		
	February	45,125 ± 9599		**0.367 ± 1.603**			** − 3.733 ± 1.630**			**5.200 ± 1.688**			**8.933 ± 0.618**		

The results were analyzed using ANOVA and Tukey’s post-hoc analysis (post-hoc). Statistical significance was set at p < 0.05, ***p < 0.001.

^a^Comparison of monthly mean values of the number of TMD patients.

^b^Comparison of monthly mean temperatures.

^c^Comparison of monthly mean values of the lowest temperature.

^d^Comparison of monthly mean values of the highest temperature.

^e^Monthly comparison of temperature differences between the highest and lowest temperatures. Temperature difference: the absolute value of the difference between the highest and lowest temperatures. 1: January, 2: February, 3: March, 4: April, 5: May, 6: June, 7: July, 8: August, 9: September, 10: October, 11: November, 12: December.

The significant results are shown in bold.

Investigation of the monthly average highest temperatures revealed that June (28.433 ± 0.899 °C), July (29.817 ± 1.408 °C), and August (30.625 ± 1.352 °C) were significantly higher, while January (2.017 ± 2.385 °C) and December (3.575 ± 1.907 °C) were significantly lower than other months (p < 0.001). The lowest average temperatures were recorded in January (2.138 ± 1.374 °C), with the highest in July (23.05 ± 1.007 °C) and August (23.65 ± 0.950 °C) (p < 0.001). The temperature difference was calculated as the absolute value subtracting the lowest temperature from the highest temperature for each month. Over the 13-year period, the largest average temperature differences were observed in April (10.033 ± 0.817 °C) and May (10.325 ± 1.0567 °C), while the smallest were in July (6.767 ± 0.885 °C) and August (6.975 ± 0.966 °C) (p < 0.001).

### Seasonal distribution of TMD patient numbers and climate temperature (2010–2022)

To investigate the seasonal variations in the number of TMD patients and climate temperature changes, the year was divided into four seasons: spring (March–June), summer (July–August), fall (September–November), and winter (December–February). The analysis of TMD patient numbers showed no distinct seasonal trends, except for a higher count in summer (49,778.89 ± 11,660.20 individuals) compared to spring (45,682.15 ± 11,100.00 individuals) (p = 0.04).

In contrast, seasonal changes in temperature were pronounced. The average highest temperatures were significantly greater in spring (20.54 ± 1.61 °C), summer (30.22 ± 1.38 °C), and fall (19.81 ± 1.21 °C) compared to winter (3.59 ± 1.99 °C) (p = 0.005). The average lowest temperatures also followed a similar pattern, being significantly higher in spring (10.72 ± 1.04 °C), summer (23.35 ± 0.98 °C), and fall (10.80 ± 1.13 °C) than in winter (− 4.72 ± 1.77 °C) (p = 0.005). Additionally, the average temperature difference was larger in spring (9.81 ± 0.93 °C) compared to summer (6.87 ± 0.93 °C) and winter (8.32 ± 0.68 °C), while fall (9.01 ± 0.90 °C) showed a larger difference than summer (p = 0.001) (Table 3).

**Table 3:** Seasonal changes of TMD patient numbers and climate temperatures over the past 13 years (2010–2022).

Season	Month	Comparison across four seasons											
		Number of TMD patients	p-value^a^	post-hoc^a^	Lowest temperature (°C)	p-value^b^	post-hoc^b^	Highest temperature (°C)	p-value^c^	Post-hoc^c^	Temperature difference (°C)	p-value^d^	Post-hoc^d^
Spring	March	45,682.15 ± 11100.09	0.04*	Spring < Summer	10.72 ± 1.04	0.005**	Spring > Winter, Summer > Winter, Fall > Winter	20.54 ± 1.61	0.005**	Spring > Winter, Summer > Winter, Fall > Winter	9.81 ± 0.93	0.001**	Spring > Summer, Spring > Winter, Summer < Fall
	April												
	May												
	June												
Summer	July	49,778.89 ± 11660.20			23.35 ± 0.98			30.22 ± 1.38			6.87 ± 0.93		
	August												
Fall	September	46,896.89 ± 11428.11			10.80 ± 1.13			19.81 ± 1.21			9.01 ± 9.01		
	October												
	November												
Winter	December	47,002.25 ± 10,592.53			− 4.72 ± 1.77			3.59 ± 1.99			8.32 ± 0.68		
	January												
	February												

The results were analyzed using ANOVA and Tukey’s post-hoc analysis. Statistical significance was set at p < 0.05, ***p < 0.001.

^a^Comparison of seasonal mean values of the number of TMD patients.

^b^Comparison of seasonal mean values of the lowest temperature.

^c^Comparison of seasonal mean values of the highest temperature.

^d^Seasonal comparison of temperature differences between the highest and lowest temperatures. Temperature difference: the absolute value of the difference between the highest and lowest temperatures.

The significant results are shown in bold.

### Temporal changes in climate temperature and TMD patient numbers over the past 13 years (2010–2022)

**Figure 5:**
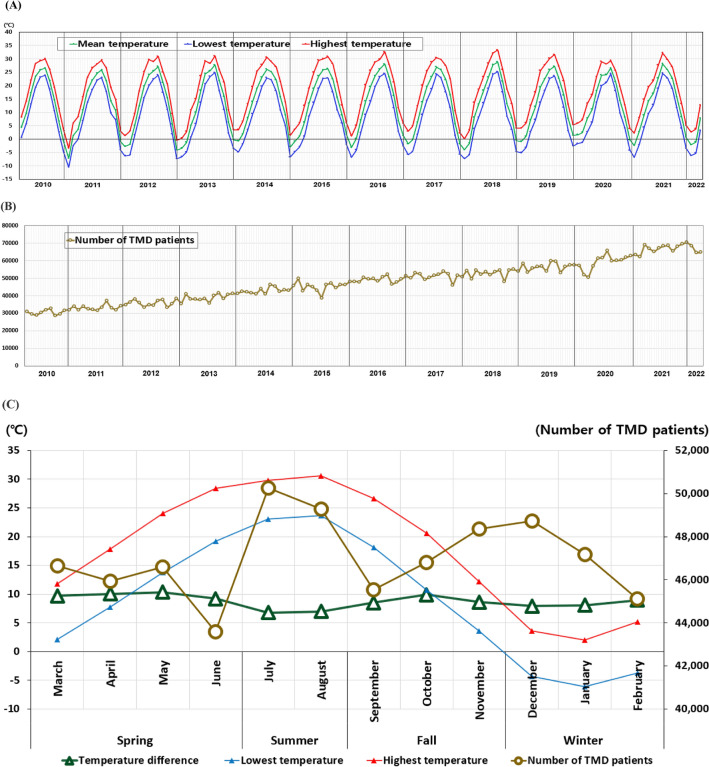
Temporal changes in climate temperature and TMD patient numbers over the past 13 years (2010–2022). (**A**) Changes in climate temperature over the past 13 years (2010–2022). (**B**) Changes in number of TMD patients over the past 13 years (2010–2022). (**C**) Seasonal changes in the number of climate temperature and TMD patients over the past 13 years (2010–2022). March–June: spring; July–August: summer; September–November: fall; December–February: winter.

Figure 5A presents a graph that continuously displays the monthly variations in climate temperatures (mean, lowest, and highest) from 2010 to 2022. Distinct temperature fluctuations within annual cycles are observed, which typically inform the delineation of seasons—spring, summer, autumn, and winter—each characterized by its seasonal attributes. However, when examining the number of TMD patients at the same time points, TMD patient numbers do not follow the pattern of climate temperatures (Fig. 5B). Instead of fluctuating within a set range, the numbers of TMD patients exhibit a unique pattern and show a progressive increase over time. Although not statistically significant, the number of TMD patients within a yearly cycle exhibits two period peaks, in July–August and November–December (Fig. 5C). The number of TMD patients typically decreases from May to June, increases from June to July, decreases again from August to September, and then continues to increase from September to December, before declining from December to February.

### The correlation between the number of TMD patients and climate temperature

**Figure 6:**
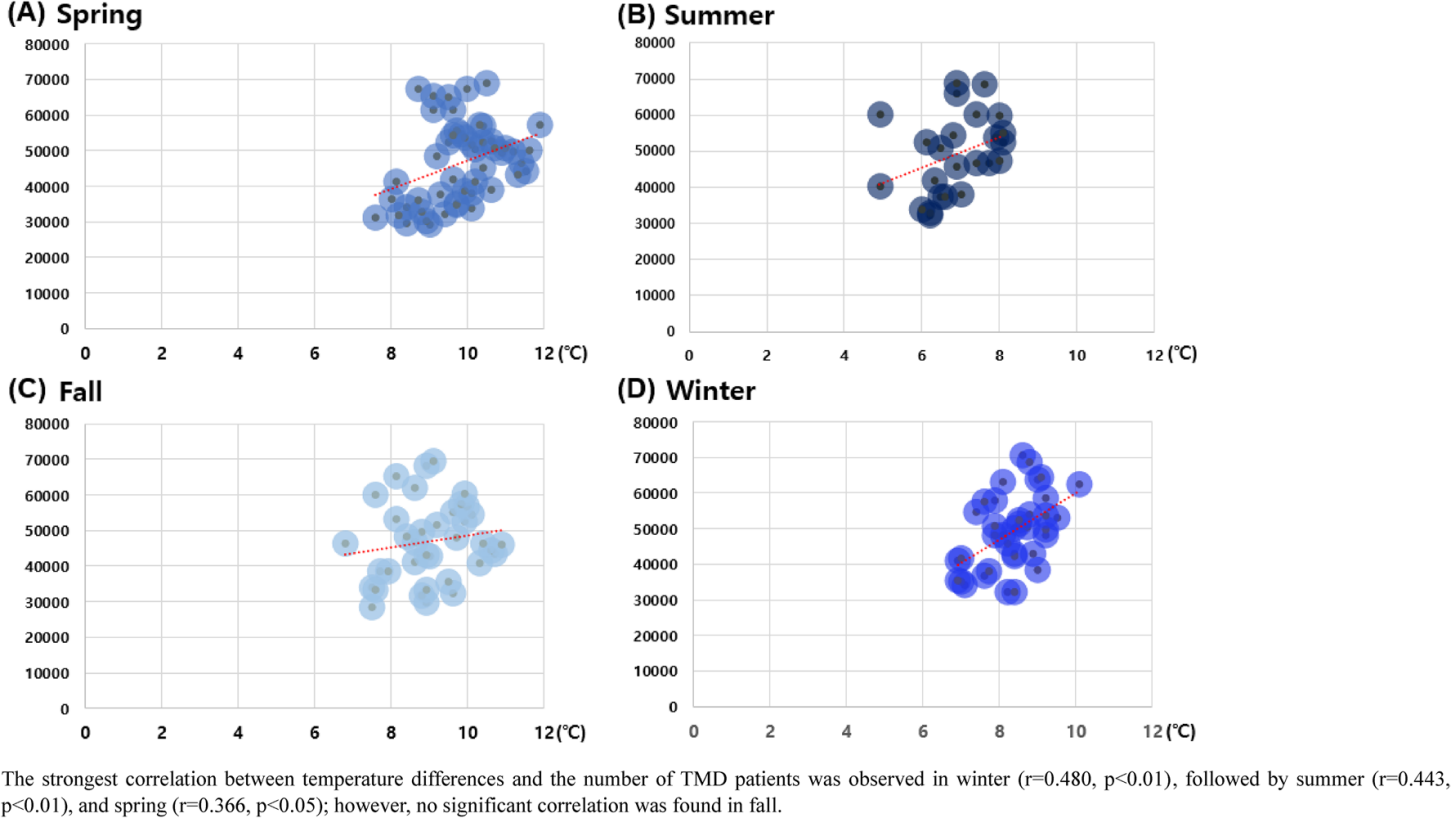
Correlation between the number of TMD patients and climate temperature difference by season (2010–2022). (**A**) Spring, (**B**) summer, (**C**) fall, and (**D**) winter. x-axis: climate temperature difference, y-axis: the number of TMD patients. The strongest correlation between temperature differences and the number of TMD patients was observed in winter (r = 0.480, p < 0.01), followed by summer (r = 0.443, p < 0.01), and spring (r = 0.366, p < 0.05); however, no significant correlation was found in fall.

Notably, temperature differences exhibited a significant correlation with the number of TMD patients in spring, summer, and winter, but not in fall (Fig. 6). The strongest correlation between temperature difference and the number of TMD patients was observed in winter (r = 0.480, p < 0.01), followed by summer (r = 0.443, p < 0.01), and spring (r = 0.366, p < 0.05). Furthermore, when considering the entire dataset without seasonal segmentation, a significant correlation was observed between TMD patients and temperature differences (r = 0.171, p = 0.04). Temperature difference exhibited a significant positive correlation with mean temperature during summer (r = 0.457, p < 0.01) and winter (r = 0.334, p < 0.05); however, this correlation was not observed in spring and fall. Both the lowest and highest temperatures demonstrated a strong correlation (r > 0.7) across all seasons, yet these temperatures did not correlate significantly with the number of TMD patients (Table 4). This crucial finding challenges the commonly held belief that lower temperatures and/or the winter season contribute to increased TMD symptoms and prevalence.

**Table 4:** The correlation between seasonal TMD patient numbers and climate temperatures.

	Spring				Summer				Fall				Winter			
	Mean temperature (°C)	Lowest temperature (°C)	Highest temperature (°C)	Temperature difference (°C)	Mean temperature (°C)	Lowest temperature (°C)	Highest temperature (°C)	Temperature difference (°C)	Mean temperature (°C)	Lowest temperature (°C)	Highest temperature (°C)	Temperature difference (°C)	Mean temperature (°C)	Lowest temperature (°C)	Highest temperature (°C)	Temperature difference (°C)
Number of patients	0.000	− 0.008	0.009	0.366*	0.335	0.144	0.315	0.443**	− 0.019	− 0.029	− 0.026	0.189	0.252	0.157	0.301	0.480**
Mean temperature (°C)		0.995**	0.996**	− 0.061		0.887**	0.921**	0.457**		0.981**	0.989**	− 0.012		0.983**	0.970**	0.344*
Lowest temperature (°C)			0.989**	− 0.107			0.700**	0.096			0.958**	− 0.124			0.932**	0.214
Highest temperature (°C)				− 0.020				0.703**				0.067				0.507**

The results were obtained using Spearman’s correlation analysis. Temperature difference: the absolute value of the difference between the highest and lowest temperatures.

Significant results are highlighted in bold. Statistical significance was set at p < 0.05, *p < 0.05, **p < 0.01, ***p < 0.001. The significant results are shown in bold.

## Discussion

This study aimed to ascertain whether the hypothesis that TMD occurs more frequently during colder winter months than warmer summer months in correlation with climate temperature is true. Accordingly, we conducted an analysis of the number of TMD patients by month and season. However, our main hypothesis that winter or low temperatures would be related to the increase in the number of TMD patients was rejected; the analysis of the HIRA open big data revealed that the difference in TMD patient numbers between summer and winter was not significant. Surprisingly, the highest average number of TMD patients was recorded during the summer months. The data revealed two periodic peaks in TMD cases throughout the year, specifically in July–August and November–December, which appears to correlate with school summer and winter vacations, as well as holidays for students and working individuals. Furthermore, our secondary hypothesis that an increase in temperature difference might be associated with a rise in TMD patient numbers was supported. By combining data from the Korean Meteorological Administration with HIRA big data, we first established a positive correlation between the temperature difference between the maximum and minimum climate temperatures and the number of TMD patients. According to Martins et al., socio-economic class does not have a significant correlation with the occurrence of TMD^25^. However, among the elderly, lower income and monthly rent fees were associated with the incidence of TMD^26^. Consequently, the sharp increase in TMD prevalence in Korea necessitates further investigation to determine if this trend reflects country-specific sociodemographic or biopsychosocial factors. The highest prevalence was noted among individuals in their 20 s, with females outnumbering males at a ratio of 1.51:1. Notably, TMD patients in the Seoul-Gyeonggi metropolitan area accounted for half of all cases nationwide.

TMD is regarded as a heterogeneous group of conditions primarily characterized by a multifactorial pathogenesis^27^. The prevailing opinion is that TMD involves both physical and psychological factors; is understood to have a multifactorial etiology encompassing parafunctional habits, bruxism, maladaptive body posture, occlusal characteristics, developmental irregularities, macro or microtrauma, excessive loading, and stress^28^. Painful TMD has been demonstrated to be biopsychosocial and multifactorial^6^. From a socio-geographic perspective, there is a noticeable differential distribution of TMD patients across administrative regions, with around 50% of TMD cases concentrated in the metropolitan area. This phenomenon likely reflects both natural population distribution patterns and the centralization of medical institutions in the capital region. Seoul and Gyeonggi province, being moderate climate zones in South Korea with densely populated areas, exhibit significantly higher numbers of TMD patients. Due to the nature of the data, aspects such as biopsychological factors like psychological issues or conditions, as well as societal aspects such as income, occupation, and marital status, were not analyzed together with this study. Consistent reports have indicated an association between lower temperatures and increased muscle pain^29,30^. As temperatures decrease, individuals with chronic pain frequently experience an exacerbation of their symptoms, resulting in increased joint discomfort, heightened muscle tension, and more severe sharp pains^31^. However, to conclusively determine the impact of seasonal and/or weather factors on TMD, a comprehensive approach that includes temperature, humidity, precipitation, wind, atmospheric pressure, and cloudiness is essential. Additionally, the analysis should incorporate advanced, cutting-edge methodologies to control for covariates and elucidate relationships.

According to the DC/TMD, common TMD can be classified into arthrogenous and myogenous TMD, and headaches can be attributed to TMD. Some factors can contribute to increased joint or muscle pain in cold weather. When temperatures drop, air pressure tends to decrease, which can directly impact joint sensitivity^32^. This might result in the expansion of soft tissues such as tendons and muscles, exerting more pressure on inflammatory arthritic joints and causing discomfort during movement^33^. Moreover, colder weather often leads to reduced physical activity and prolonged indoor stay. Extended periods of inactivity can lead to muscle weakening and decreased joint flexibility, contributing to muscle stiffness and an increased risk of painful cramps^34^. Seasonal affective disorder, which is prevalent during colder months, may influence pain perception owing to its impact on mood^35^. Reduced daylight exposure and brightness during winter may exacerbate psychological aspects^36^. Additionally, individuals with heightened nerve sensitivity may experience amplified muscle and joint pain in response to cold temperatures, as cold weather tends to negatively affect nerve conduction, intensifying existing nerve-related issues^37^. In a study conducted in Taiwan, patients with migraines were asked to keep a headache diary for one year, and 51.5% were affected by weather changes, but 48.5% were not^38^. However, the effects of the weather on headaches attributed to TMD have not yet been investigated.

Approximately 67% of patients with joint pain, including osteoarthritis and rheumatoid arthritis, believe that weather factors worsen their symptoms; however, external weather conditions do not significantly affect the daily symptoms of arthritis^39^. According to Tsai et al., an abrupt temperature change is a triggering factor that increases arthralgia^40^. This can serve as evidence to support the results of this study, showing an increase in temperature change and the number of patients with TMD. In the most recently reported meta-analysis, levels of musculoskeletal pain were higher in cold countries and lower in countries with warm climates; however, heterogeneity in patient composition and a lack of studies hindered the valid synthesis or analysis of risk scales^16^. In a recent study conducted in Morocco, symptom severity in patients with rheumatoid arthritis was not significantly affected by seasonal changes in temperature^41^. However, Morocco is a country with a rainy season and a dry season, and the climate alternates between warm and humid (average 15 °C) in the rainy season and hot and dry (average 28 °C) in the dry season. Therefore, the situation differs from that in Korea, where the spring, summer, fall, and winter are distinct. Few studies have investigated changes in TMD prevalence or symptom severity according to seasonal or temperature changes. TMD is a major musculoskeletal disorder affecting the orofacial region and is one of the most common conditions causing severe facial pain^5,6^. Additional research is needed to determine the myth or reality of changes in TMD symptoms depending on weather or season.

An essential aspect of this study was the utilization of robust national data spanning a 13-year period to conduct statistical analyses of patients seeking treatment for TMD at hospitals. Notably, our findings align with prior research on TMD prevalence, indicating a peak occurrence among individuals in their 20 s^42,43^. However, according to a prospective cohort study in the United States, the prevalence of TMD tends to increase with age, with the highest prevalence in the 35–44 years age group^44^. In this study, a noteworthy departure was observed in the mid-to-late teenage demographics, exhibiting substantial prevalence, unlike the patterns observed in other studies. Contrary to prevailing trends, this age group (mid-to-late teenage) demonstrated a heightened prevalence, potentially linked to the social and psychological stress experienced by Korean middle and high school students^45^. Academic pressure, interpersonal relationships, and developmental challenges during this phase may have contributed to the atypical prevalence. Additionally, a notable observation was the significant occurrence of TMD among the elderly, particularly those aged over 65 years and notably over 80 years.

In the analysis of HIRA data, inpatient treatment accounted for only 0.1% of TMD patients, while outpatient treatment comprised 99.9%. This indicates that treatments based in outpatient settings were conducted 996 times more frequently than those involving procedures or surgeries requiring hospital admission. The vast discrepancy between these two treatment strategies is primarily due to the focus of this study on general and typical TMD cases (4-digit KCD code: K07.6), explicitly excluding other conditions and diseases that could present TMD-like signs and symptoms. Generally, the current clinical management options for typical TMD prioritize non-invasive, and minimally invasive^46^. As part of conservative outpatient treatment, patient education and cognitive behavior therapy are typically implemented first^15^. In cases of acute jaw locking, manual manipulation is used to adjust the relationship between the mandible and the articular disc^47^. For TMD pain management, appropriate pharmacological treatments such as nonsteroidal anti-inflammatory drugs, steroids, muscle relaxants, and tricyclic antidepressants are administered based on the source of the pain^48^. Injection therapies for TMD include direct administration of steroids and/or dexamethasone into the TMJ space and botulinum toxin injections into the masticatory muscles^15,49^. For the treatment of myofascial pain, trigger point injections can be employed^50^. Orthopedic stability is often restored through TMJ stabilization and muscle relaxation, with treatments including the use of stabilization splints^15,51^. Additionally, adjunct therapies such as moist heat packs or ultrasound therapy can be employed^52^. These treatments are typically utilized either individually or in combination. When conservative treatments are deemed ineffective, surgical interventions, such as arthroscopy and open-joint surgery, are considered.

One limitation of this study is the lack of a detailed examination of TMD diagnosis, symptom severity, duration, and treatment specifics among patients with TMD. While acknowledging the significant influence of psychological factors on the physical factors of signs and symptoms of TMD, this study did not incorporate these psychological aspects. During the transition from fall to winter, colder temperatures and reduced sunlight durations may trigger mood alterations or depression in certain individuals^53^. TMD is defined as chronic pain, and weather and temperature factors that affect mood and psychology have been considered in chronic pain^54,55^. Additional comprehensive investigations and analyses of these elements are required.

For the first time, we have examined the trends in TMD prevalence over the past 13 years in South Korea. In this country, the National Health Insurance (NHI) covers the majority of the population, with most citizens enrolled in this program. According to the National Health Insurance Service, as of 2021, NHI enrollees accounted for over 97% of the total population, with the remainder being beneficiaries of Medical Aid, a welfare program for low-income individuals. When NHI enrollees visit hospitals, these visits are automatically recorded in the HIRA through the medical billing process. Therefore, although this study did not investigate TMD among the lowest economic strata, it was possible to ascertain the prevalence of TMD among the vast majority of the population. Additionally, we investigated fluctuations in TMD patient numbers concerning seasons and temperature variations in Korea, utilizing HIRA healthcare big data in conjunction with data from the Korea Meteorological Administration. Consequently, we found that neither low absolute temperatures nor the winter season had a significant relationship with an increase in TMD patient numbers; rather, the temperature difference between the highest and lowest temperatures was a more decisive factor in South Korea. These findings should be a key consideration when examining seasonal trends in TMD prevalence in South Korea. Furthermore, this research will provide valuable information and insights into the impact of temperature differences on TMD for clinicians and patients worldwide.

## Data Availability

The datasets used and/or analyzed in the current study are available from the corresponding author upon reasonable request. Korean patients’ health statuses, medical providers, medical expenses, utilization rates, and summary statistics related to medical services were made public through the Healthcare Big Data Open System (http://opendata.hira.or.kr). Monthly temperature data for the past 13 years, spanning from 2010 to 2022, were obtained from the National Climate Data Center of the Korea Meteorological Administration (https://data.kma.go.kr). The total population of South Korea for the same period was sourced from the online National Population Statistics portal (https://jumin.mois.go.kr).
